# Alignment of Mitotic Chromosomes in Human Cells Involves SR-Like Splicing Factors Btf and TRAP150

**DOI:** 10.3390/ijms18091956

**Published:** 2017-09-12

**Authors:** Sapna Varia, Divya Cheedu, Michael Markey, Keshia Torres-Shafer, Vishnu Priya Battini, Athanasios Bubulya, Paula A. Bubulya

**Affiliations:** 1 Biomedical Sciences Ph.D. Program, Wright State University, Dayton, OH 45435, USA; sapnavaria@gmail.com; 2 Masters of Biological Sciences Program, Wright State University, Dayton, OH 45435, USA; cheedu.3@wright.edu (D.C.); torres-shafer@wright.edu (K.T.-S.); vish.battini@gmail.com (V.P.B.); 3 Center for Genomics Research, Wright State University, Dayton, OH 45435, USA; michael.markey@wright.edu; 4 Department of Biological Sciences, Wright State University, Dayton, OH 45435, USA

**Keywords:** serine-arginine-rich (SR) proteins, Btf, TRAP150, pre-mRNA splicing, mitosis, cell cycle

## Abstract

Serine-arginine-rich (SR) or SR-like splicing factors interact with exon junction complex proteins during pre-mRNA processing to promote mRNA packaging into mature messenger ribonucleoproteins (mRNPs) and to dictate mRNA stability, nuclear export, and translation. The SR protein family is complex, and while many classical SR proteins have well-defined mRNA processing functions, those of other SR-like proteins is unclear. Here, we show that depletion of the homologous non-classical serine-arginine-rich (SR) splicing factors Bcl2-associated transcription factor (Btf or BCLAF) and thyroid hormone receptor-associated protein of 150 kDa (TRAP150) causes mitotic defects. We hypothesized that the depletion of these SR-like factors affects mitosis indirectly through an altered expression of mitotic checkpoint regulator transcripts. We observed an altered abundance of transcripts that encode mitotic regulators and mitotic chromosome misalignment defects following Btf and/or TRAP150 depletion. We propose that, in addition to their previously reported roles in maintaining mRNA distribution, Btf and TRAP150 control the abundance of transcripts encoding mitotic regulators, thereby affecting mitotic progression in human cells.

## 1. Introduction

The efficient expression of protein-coding genes requires transcription by RNA polymerase II, with co-transcriptional pre-mRNA 5′ capping, splicing, and 3′ end cleavage and polyadenylation. In human cells, pre-mRNA processing factors localize in small domains of the nucleus called nuclear speckles [1]. A proteomic analysis of nuclear speckles has revealed the presence of at least 180 proteins, many of which are involved in pre-mRNA processing and are enriched with pre-mRNA splicing factors, small nuclear ribonucleoprotein particles (snRNPs), serine-arginine rich proteins (SR proteins), and the large subunit of RNA polymerase II [2,3]. Two SR-like proteins, called Btf and TRAP150, were among 33 novel proteins discovered a decade ago during a proteomic analysis of purified nuclear speckles [2,3]. SR proteins have a wide variety of activities serving as regulators of splicing, mRNA export, mRNA stability, and quality control [4]. Btf has been previously described as a Bcl2-associated transcription factor, a nuclear speckle protein with an arginine-serine-rich (RS) domain at its N-terminus [2,3,5]. Thyroid hormone receptor associated protein of 150 kDa (TRAP150) was first identified as a component of the nuclear receptor TRAP complex [6,7]. Interestingly, Btf and TRAP150 are homologous proteins that have similar localization patterns and share a high degree of similarity in their primary sequence, both proteins having an amino-terminal RS domain as their only known sequence motif [2,3,8].

Our previous work demonstrated that the depletion of Btf, but not TRAP150, caused an accumulation of polyadenylated RNA in the cytoplasm of HeLa cells and pointed toward distinct functions of Btf and TRAP150 in the global regulation of mRNA cellular distribution [8]. In this report, we show the metaphase chromosome misalignment and alteration of key mitotic transcripts that is required for cell cycle progression following the depletion of Btf and/or TRAP150. A lack of co-localization of Btf or TRAP150 with proteins in any mitotic structure suggests an indirect role of Btf and/or TRAP150 in cell cycle progression. Here, we show an altered abundance of mitotic checkpoint transcripts upon the depletion of Btf/TRAP150 to explain the observed mitotic defects. To the best of our knowledge, this is a novel function of the splicing factors Btf and TRAP150 in cell cycle regulation. Btf and TRAP150, therefore, have overlapping functions in human cells with regard to cell cycle regulation, in contrast to their having distinct roles in the regulation of mRNA distribution.

## 2. Results

### 2.1. Depletion of Btf and/or TRAP150 Results in Mitotic Defects

**Figure 1:**
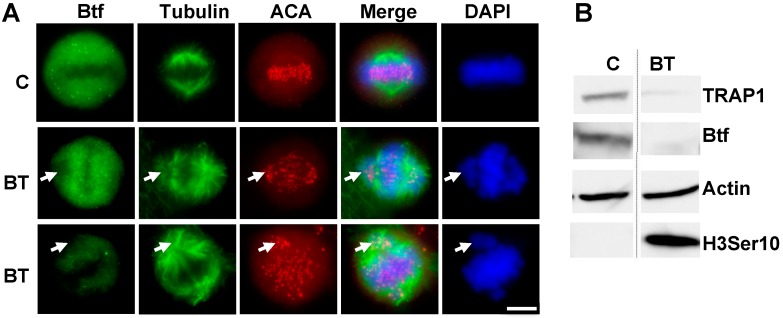
Depletion of Btf and TRAP150 causes chromosome misalignment defects. (**A**) HeLa cells were transfected with siRNA duplexes targeting Btf and TRAP150 mRNA (BT) or control siRNA duplex (C) and processed for immunolocalization of Btf, beta-tubulin and ACA 72 h post-transfection; DNA was stained with DAPI. Merge is shown for beta-tubulin and DAPI. Arrows indicate misaligned chromosomes. Scale bar, 5 μm; (**B**) Immunoblot of HeLa cell extracts 72 h following treatment with control siRNA (siGENOME2) or siRNA duplexes targeting Btf and TRAP150 (BtfSi1 and TRAPSi4) confirmed Btf and TRAP150 protein depletion with actin as a loading control. Anti-H3Ser10P detected phosphorylated histone H3Ser10. Scale bar = 5 µm.

DAPI staining revealed a misalignment of metaphase chromosomes after the treatment of HeLa cells with respective sets of siRNA duplexes targeting Btf and TRAP150 at concentrations known to efficiently reduce Btf and/or TRAP150 mRNA and protein levels (Figure 1 and Figure S1, and Ref. [8]). Btf and TRAP150 siRNA treatment resulted in chromosome misalignment defects (Figure 1, arrows) regardless of having a less (middle row) or a more (bottom row) efficient depletion across the coverslip as monitored by Btf immunofluorescence. This is consistent with the idea that the absence of Btf and/or TRAP150 interferes with the progression of cells through mitosis. As expected, immunoblotting showed an increased phosphorylation of histone H3Ser10, indicating a significantly higher abundance of mitotic cells following Btf and TRAP150 depletion (Figure 1B; lane BT).

### 2.2. Btf and TRAP150 Regulate Cell Cycle Progression in HeLa Cells

**Figure 2:**
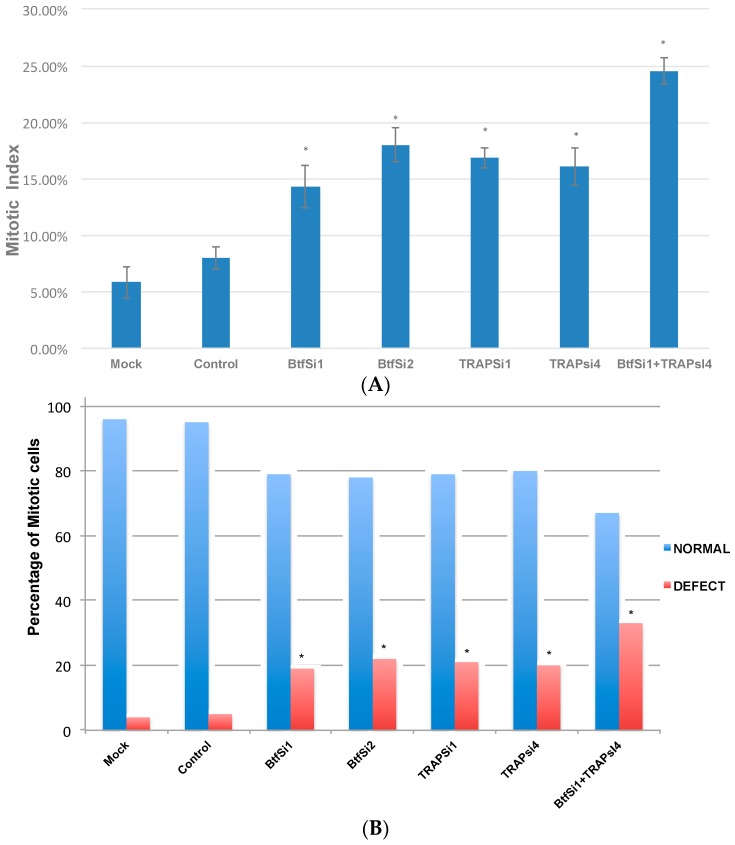
Absence of Btf and/or TRAP150 alters cell cycle distribution. (**A**) Mitotic index was scored in three replicate experiments for HeLa cells treated with mock conditions, control siRNA duplexes, or siRNA duplexes; (**B**) In a separate experiment, 100 metaphase cells were scored as “normal” or “defective” regarding metaphase chromosome alignment. Asterisks in both tables indicate significant difference from control (*p*-value ≤ 0.001).

To further study the mitotic defects in Btf/TRAP 150-depleted samples, mitotic distribution and mitotic index were calculated following Btf and/or TRAP150 RNAi in HeLa cells. Microscopy was used to score 500 mitotic cells in each sample from three replicate experiments. These were categorized according to mitotic stages as well as for normal versus defective chromosome arrangement (Figure 2). The mitotic index was significantly higher in the depleted samples as compared to control samples (Figure 2A). The mitotic index was 7% in cells treated with control siGENOME2 siRNA duplexes, and increased to 15.2% in cells treated with Btf Si1 siRNA duplexes, 18.7% in cells treated with Btf Si2 siRNA duplexes, 17.9% in cells treated with TRAP150 Si1 siRNA duplexes, and 15.2% in cells treated with TRAP150 Si4 duplexes. The combined depletion of Btf and TRAP150 showed an even higher mitotic index of 25.62%. Interestingly, although mitotic cells were more abundant following knockdown of Btf and TRAP150, there was a similar distribution of mitotic stages among the different samples (Figure S2). Importantly, the number of metaphase cells displaying chromosome misalignment defects was significantly higher in Btf- and TRAP150-depleted cells than in control cells or in cells after depletion of either Btf or TRAP150 (Figure 2B).

Our results demonstrated that a reduction in the levels of Btf, TRAP150, or both Btf and TRAP150 (Figure 1A) causes a misalignment of chromosomes at metaphase. A reduction in the levels of different SR proteins or splicing factors has been previously implicated to cause cell cycle defects [9] or genomic instability [10,11]. The results in Figure 1 and Figure 2 suggest that Btf and TRAP150 play an important role during normal cell cycle progression particularly related to proper metaphase chromosome alignment. A flow cytometry analysis (Figure S1) confirmed that G2/M phase cells increased from 12% in controls (non-targeting siGENOME2 siRNA duplexes) to 21% or 23% in cells depleted with two separate Btf siRNAs, or to 21% or 22% in cells depleted with two separate TRAP150 siRNAs. In addition, G2/M phase cells increased to 37% in HeLa cells following the depletion of both Btf plus TRAP150 (BT), providing evidence for their additive functions. Previous studies [8] have shown that TRAP150 depletion results in an upregulation of Btf, and that Btf depletion results in the upregulation of TRAP150. We therefore considered that the depletion of one protein might be compensated by the upregulation of the other, and that co-depletion of both Btf and TRAP150 would increase the incidence of metaphase chromosome misalignment. Consistent with this idea, the co-depletion of Btf and TRAP150 further increased the misalignment of chromosomes at metaphase as compared to the single depletion of either protein (Figure 2). Interestingly, increased numbers of 8N cells indicate that mitosis may be incomplete in these conditions (Figure S1). Taken all together, our data shows that Btf and/or TRAP150 depletion interferes with the cell cycle and leads to metaphase chromosome misalignment and a possible failure of mitotic progression.

### 2.3. Btf and/or TRAP150 Depletion Alters Expression of Cell Cycle Regulator Transcripts

Exhaustive localization studies were done to show that neither Btf nor TRAP150 localize to specific mitotic structures (see also Ref. [8]). We therefore hypothesized that Btf/TRAP150 indirectly regulate chromosome alignment and potentially the spindle assembly checkpoint by controlling the abundance and/or processing of chromosomal passenger protein transcripts. Proper alignment of chromosomes during metaphase is regulated by the spindle assembly checkpoint (SAC). The SAC ensures proper chromosome segregation by preventing anaphase onset until all chromosomes are properly attached to microtubules that align chromosomes at the metaphase plate. Since a reduced expression of these proteins has a similar effect on mitotic progression, we hypothesized that specific cell cycle regulator transcripts might be affected by Btf or TRAP150 depletion. We used a microarray analysis to determine global transcript changes following the knockdown of Btf or TRAP150. The expression of transcripts for Aurora-B, CENP-E, CENP-F, BUB1, BUB1B, CDK1, CDC20, and MAD2L1 was upregulated upon Btf and/or TRAP150 depletion (Table 1).

**Figure 3:**
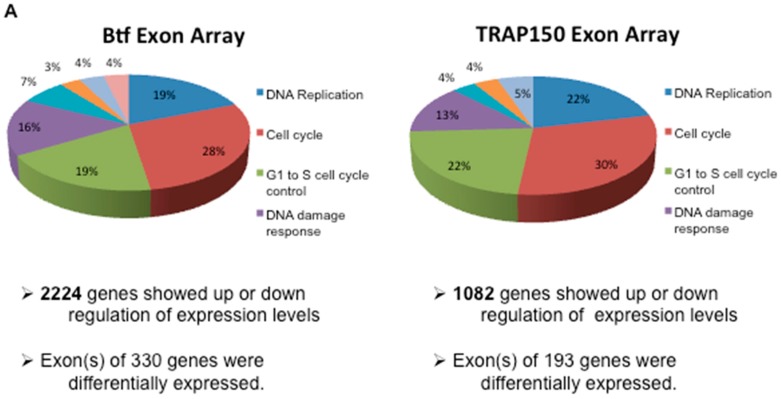

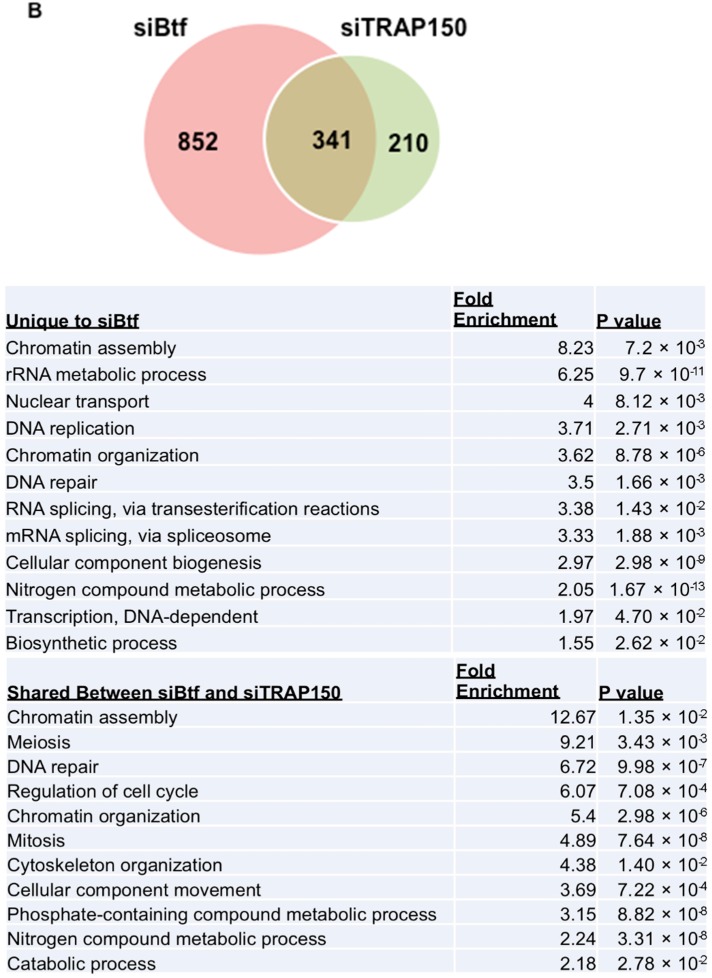
Pathways affected in Btf and TRAP150 depleted cells. (**A**) Overlapping pathways and total transcript and splicing changes between Btf- and TRAP150-depleted samples. Unique to Btf were fluoropyrimidine activity (7%), benzo(a)pyrene metabolism (4%), and nucleotide metabolism (4%). Unique to TRAP150 were the alpha 6 beta 4 signaling pathway (5%) and the mitochondrial long chain fatty acid beta oxidation pathway (4%); (**B**) Btf and TRAP150 share transcriptional effects on a subset of genes. Differentially expressed genes (Benjamini-Hochberg -adjusted *p*-values < 0.05, fold change > 2) for siBtf and siTRAP150 were compared. A set of 341 shared genes were differentially expressed in response to siBtf or siTRAP150. There were 852 differentially expressed genes unique to siBtf, but only 210 in siTRAP150. These gene lists were submitted for gene ontology analysis. The gene ontology analysis was performed using the PANTHER Overrepresentation Test (Ref. [20]) (release 20170413) using all Homo sapiens genes as the reference list and the PANTHER GO-Slim biological process as the annotation data set. The genes uniquely regulated by siBtf were enriched for genes involved in chromatin assembly, RNA splicing, and other processes, while the list of genes unique to siTRAP150 has no significantly over-represented biological processes. However, the genes regulated by both Btf and TRAP150 were involved in chromatin assembly, meiosis, DNA repair, and other processes as indicated.

The cellular pathways affected by Btf versus TRAP150 depletion overlapped extensively; however, some pathways were unique (Figure 3). Cell cycle was the most significant pathway affected in both the Btf- and the TRAP150-depleted samples, followed by G1/S cell cycle control, DNA replication, DNA damage response, and homologous recombination. Lists of the genes whose expression was most significantly up or down in Btf- or TRAP150-depleted cells appear in Tables S1–S4.

**Figure 4:**
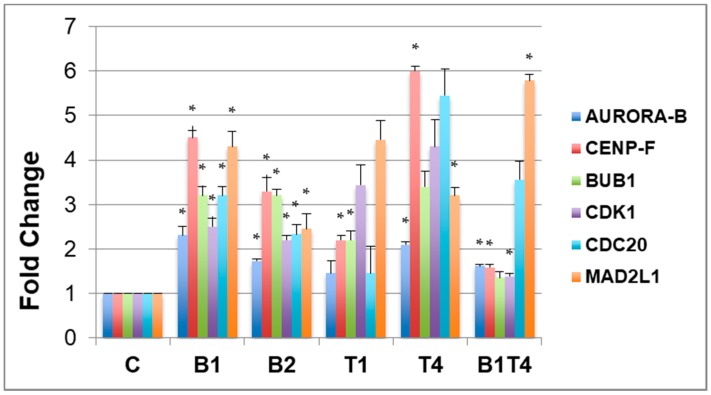
Validation of upregulation for major checkpoint protein transcripts upon Btf and/or TRAP150 depletion. RNA samples from HeLa cells treated with control siGENOME2 (C), Btf siRNA duplexes (B1, B2), TRAP150 siRNA duplexes (T1, T4), or Btf + TRAP150 siRNA duplexes (B1T4) were used for RT-qPCR analysis to validate alterations in transcripts that encode mitotic regulator proteins. * *p*-Values ≤ 0.05 for each respective gene compared to its level in control samples (e.g., aurora-b in sample B1T4 compared to Aurora-B in sample C).

Immunoblots and corresponding densitometry plots show the expression levels of Btf or TRAP150 in each of the three replicate samples (Figure S3) used for the transcript abundance assays by RT-qPCR (Figure 4). These RT-qPCR data validate that mitotic checkpoint transcripts are affected by Btf and/or TRAP150 depletion, supporting our hypothesis that the mitotic chromosomal defects observed upon Btf and TRAP150 depletion are a consequence of the misregulation of mitotic checkpoint gene transcripts in the absence of Btf and TRAP150. Our studies, therefore, suggest that Btf and TRAP150 promote mitotic progression in human cells by maintaining proper expression levels of key mitotic regulator transcripts.

### 2.4. Mitotic Checkpoint Protein Aurora-B Localizes to Misaligned Chromosomes at Metaphase in Btf- and/or TRAP150-Depleted Cells

**Figure 5:**
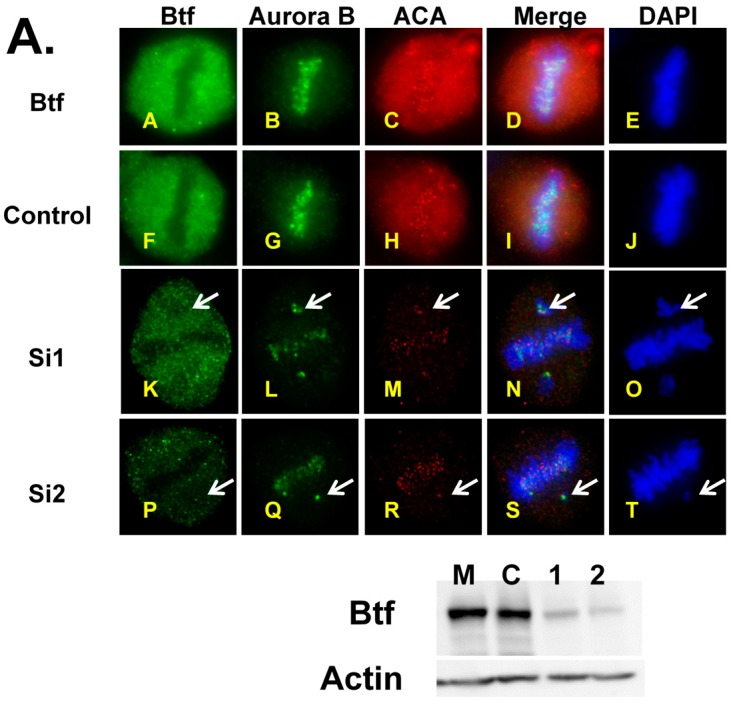

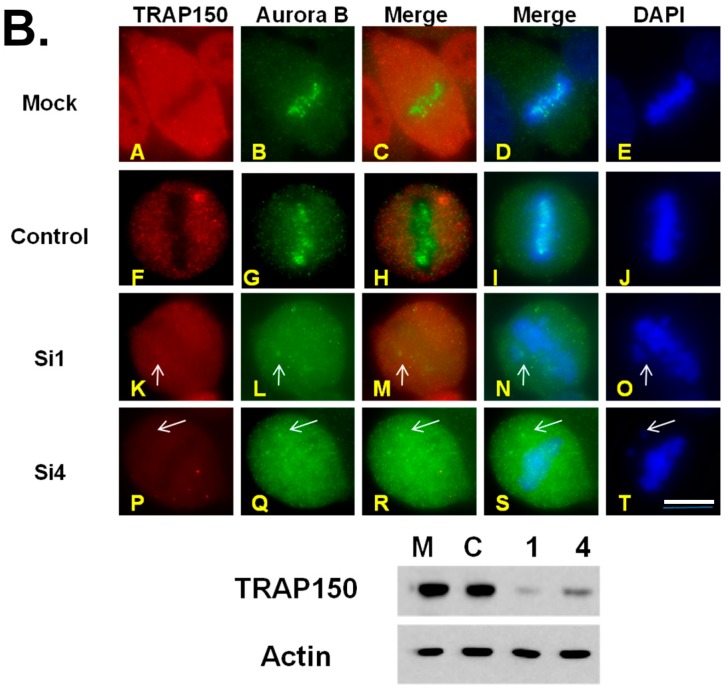
Aurora B localizes to misaligned chromosomes at metaphase in Btf-depleted cells. HeLa cells were treated with mock conditions (siRNA buffer), siRNA against control luciferase, Btf siRNA duplexes (**A**), or TRAP150 siRNA duplexes (**B**). Seventy-two hours (72 h) post-transfection, the cells were processed for immunolocalization of Btf/TRAP150, Aurora B (B,G,L,Q), and ACA (C,H,M,R). DNA was stained with DAPI. Arrows indicate misaligned chromosomes. Immunoblot confirmed the depletion of Btf/TRAP150, and β-Actin was used as a loading control. Scale bar = 5 µm.

Presumably, even one unattached kinetochore is sufficient to prevent a progression to anaphase due to spindle checkpoint activation [12,13]. During early mitosis, mitotic checkpoint proteins such as Aurora-B, Mad2, Mad1, BubR1, and BUB1 maintain increased abundance at unattached kinetochores compared to attached kinetochores subjected to proper spindle tension [14,15,16,17,18,19]. We tested if mitotic checkpoint protein Aurora B (a mitotic checkpoint protein that oversees proper kinetochore–microtubule attachments) was localized at kinetochores of misaligned chromosomes following Btf and TRAP150 depletion. If this mitotic checkpoint is active, then the mitotic checkpoint protein Aurora B should localize to the kinetochores of misaligned chromosomes. HeLa cells depleted of either Btf or TRAP150 were immunolabeled for either Btf or TRAP150 and Aurora B (Figure 5).

Immunolabeling showed that Aurora B localized to kinetochores of misaligned chromosomes in depleted cells. An immunofluorescence analysis showed higher intensity Aurora B labeling at kinetochores of misaligned chromosomes than on kinetochores of the aligned chromosomes at metaphase in Btf- and/or TRAP150-depleted cells. Despite the misregulation of Aurora B transcript level following Btf or TRAP150 depletion, the abundance of Aurora B protein at misaligned chromosomes suggests that its spindle assembly checkpoint activity is maintained under such conditions. While the specific mechanism underlying defective chromosome alignment in Btf/TRAP150-depleted cells remains to be elucidated, our data point to the misregulation of cell cycle regulator transcripts that ultimately control cell division outcomes.

### 2.5. Mitosis Is Delayed or Incomplete in the Absence of Btf and/or TRAP150

**Figure 6:**
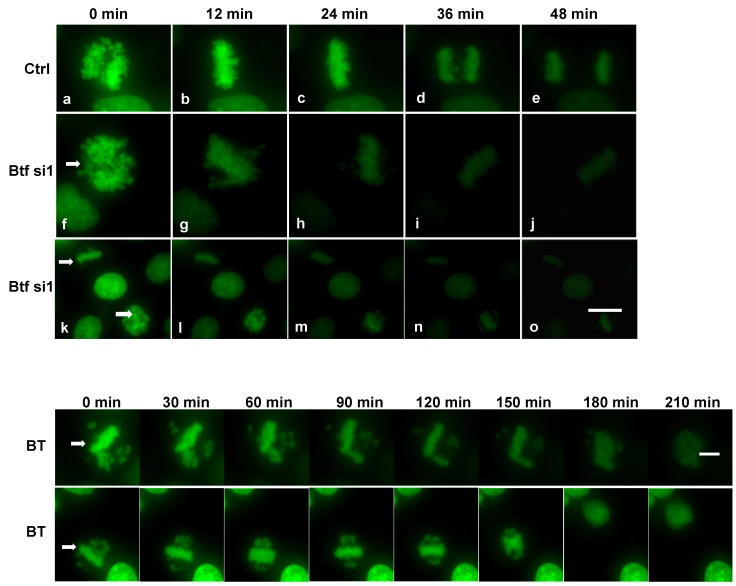
Live imaging of mitotic cells following Btf or Btf and TRAP150 depletion. Time-lapse imaging of HeLa cells stably expressing H2B-YFP initiated 72 h post siRNA transfection with control siRNA (**a**–**e**) or Btf si1 (one example shown in (**f**–**j**) and two additional examples in (**k**–**o**)) or BtfSi1 and TRAP150si4 (BT). Cells having chromosome misalignment defects upon reaching metaphase are indicated with arrows. Scale bar = 5 µm.

Our data support the idea that the misregulation of checkpoint transcripts, such as for the mitotic checkpoint protein Aurora B, does not necessarily affect Aurora B protein localization to the misaligned chromosomes. Most importantly, these data indicate that misaligned chromosomes are most likely unattached to spindle microtubules in Btf-depleted (or TRAP150-depleted) cells, a checkpoint mechanism that would be expected to arrest cells in metaphase to prevent aneuploidy. Nonetheless, we wished to determine the fate of misaligned chromosomes by time-lapse microscopy in HeLa cells stably expressing H2B-YFP transfected either with control siRNA duplexes, Btf siRNA duplexes, or TRAP150 siRNA duplexes (Figure 6). After 72 h, siRNA-treated cells were placed in a live cell chamber and 10 movies were obtained per experimental condition. The results showed that the average total time cells were in mitosis was longer in depleted cells than the control cells when either Btf or TRAP150 alone were depleted (Figure 6 and Figure S4). Upon the depletion of BTF and TRAP150, cells often failed to properly complete mitosis, as chromosomes typically would not segregate (Figure 6, BT). In such cases, chromosomes were observed in a single nucleus following incomplete mitosis (Figure 6, bottom row, 210 min) that supports our hypothesis for an increased abundance of 8N cells (Figure S1).

## 3. Discussion

Our data show that the absence of Btf and/or TRAP150 causes mitotic chromosome misalignment that interferes with mitotic progression in human cells. We observed high functional overlap in gene families regulated by Btf and TRAP150, particularly for transcripts encoding cell cycle regulators. Previous studies showed that Btf and TRAP150 have different functions in regulating mRNA distribution [8]. The new information gained in the present study shows that there is more functional overlap than previously appreciated for Btf and TRAP150 with regard to transcript targets. It is also interesting to note that co-depletion of Btf + TRAP150 indicated a less robust difference in expression, although still significantly different from controls, for certain transcripts. Perhaps the targets that remain most highly misregulated following Btf + TRAP150 depletion will have the greatest impact leading to the observed chromosome misalignment defects. In the future, it will be interesting to test this idea, to learn if any one or a subset of these regulators is the key player as a Btf/TRAP150 transcript target, as well as precisely how misregulation of its expression affects the underlying mechanisms of metaphase chromosome alignment. 

## 4. Materials and Methods

### 4.1. Cell Culture and RNAi

HeLa cells were grown in Dulbecco’s Modified Eagle Medium (DMEM; Hyclone, Thermoscientific, UT, USA) supplemented with 10% fetal bovine serum (FBS; Hyclone, Thermoscientific, UT, USA) and 1% penicillin/streptomycin (Invitrogen, Carlsbad, CA, USA). For RNAi, 1 × 10^5^ HeLa cells were plated in antibiotic-free medium. After 24 h, siRNA was done using Oligofectamine (Invitrogen, Carlsbad, CA, USA) alone (mock) or mixed with a non-targeted siRNA duplexes to luciferase (60 pmol; control; catalog No. D-001210-02; Dharmacon RNA Technologies) or with 60 pmoles Btf siRNA duplexes (siGENOME 1 (D-020734-01) target sequence: GAACAUAGUACUCGGCAAA; siGENOME2 (D-020734-02) target sequence: GGAAUGAGACGACCUUAUG; Dharmacon RNA Technologies, Lafayette, CO, USA) or 60 pmoles TRAP150 siRNA duplexes (siGENOME 1 (D-019907-01) target sequence: GGUAUAAGCUCCGAGAUGAUU and siGENOME 4 (D-019907-04) target sequence: GUUGAUCUCCGCCUUGAUAUU). For double knockdown of Btf and TRAP150, the cells were transfected with BtfSi1 oligos (60 pmoles) and TRAPSi4 (60 pmoles)). Cell cycle regulator transcript abundance was not substantially different in RNA samples isolated from mitotic HeLa cells following synchronization (compared to unsynchronized cells); we therefore have ruled out any concern that an increased abundance of mitotic cells in Btf/TRAP150-depleted cells had simply enriched for a specific subset of transcripts.

### 4.2. Immunofluorescence

Cells grown on coverslips were washed once with 1× PBS and fixed with 2% formaldehyde (in 1× PBS) for 15 min followed by three washes with 1× PBS for 5 min each. The cells were then permeabilized for 5 min with 0.2% TritonX-100 and washed three times with 0.5% normal goat serum (NGS, Gibco-Thermo Scientific, Waltham, MA, USA) in 1× PBS. The coverslips were then incubated with primary antibodies (Btf (WU10; 1:2500), TRAP150 (1:1000, Bethyl catalog number A300-956A)) diluted in 0.5% NGS-1 × PBS cell side up in a humidified chamber for one hour at room temperature. After three washes with phosphate buffered saline with 0.5% normal goat serum (PBS/0.5% NGS), the cells were incubated in appropriate secondary antibodies conjugated with Texas Red, Cy5, or FITC (1:500; Jackson Immunoresearch Laboratories, West Grove, PA, USA). DNA was stained with DAPI (10 μg/mL). Finally, the coverslips were mounted in anti-fade polyphenylenediamine medium and sealed with a clear nail polish. Cells were imaged using a DeltaVision RT microscope equipped with a 60x objective (1.4 numerical aperture; Olympus, Tokyo, Japan), and softWoRx 2.50 software (DeltaVision by Applied Precision/GE Healthcare, Issaquah, WA, USA). Raw images were displayed as volume projections. Mitotic stage was scored according to the distinctive appearance of chromatin at prophase, metaphase, anaphase, and telophase.

### 4.3. Cell Extract Preparation and Immunoblotting

Cells were washed briefly with 1× PBS and scraped in 1× PBS and collected into tubes. The cells were then resuspended in 1× Laemmli buffer (0.01M Tris (pH 6.8), 1% SDS, 10% glycerol, and 0.1% β-mercapthoethanol). Equal amounts of protein were loaded to 7% SDS-PAGE and transferred to a nitrocellulose membrane for immunoblotting (IB). The membrane was then blocked with 5% not-fat dry milk in 1× PBS + Tween 20 (0.5%). Rabbit anti-TRAP 150 (Bethyl, 1:1000), WU10 (Ref. [8]; 1:1000), or anti-β-actin (1:3000, Sigma-Aldrich, St. Louis, MO, USA, Cat. No. A5441) were used as primary IB antibodies, followed by secondary horseradish peroxidase (HRP)-conjugated donkey anti-mouse or HRP-conjugated donkey anti-rabbit IgG (Jackson ImmunoResearch Laboratories, West Grove, PA, USA) used at 1:25,000. Enhanced chemiluminescence Western blotting substrate (Thermo Scientific, Waltham, MA, USA) was used to detect HRP. For the cell cycle progression experiments, the following antibodies were used. Mouse anti-tubulin (Sigma, St. Louis, MO, USA 1:2500 for IF or WB), mouse anti-beta-actin (Sigma, 1:3000 for IF or WB), human anti-ACA antibody (Abcam, Cambridge, MA, USA, 1:3000 for IF), mouse anti-Aurora-B (Abcam, Cambridge, MA, USA, 1:100 for IF and WB), and mouse anti- phospho-H3 antibody (Abcam, Cambridge, MA, USA, 1:100 for WB).

### 4.4. Flow Cytometry Analysis

HeLa cells transfected with control siRNA duplexes, two different sets of siRNA duplexes against Btf (BtfSi1, BtfSi2), two different sets of siRNA duplexes against TRAP150 (TRAPSi1, TRAPSi4), or with BtfSi1 and TRAPSi4 (B1T4) for the double depletion studies were processed 72 h post transfection for cell cycle analysis by flow cytometry. The cells were collected by centrifugation at 1500 revolutions per minute (RPM) for 2 min at 4 °C. The pellet of cells was then resuspended in 200 μL of ice-cold 1× PBS. One milliliter (1 mL) of ice-cold ethanol was added by slow vortexing to fix the cells. The cells were then placed overnight at −20 °C. The next day, the cells were centrifuged at 1500 RPM for 5 min to remove ethanol and washed with ice-cold 1× PBS at 4 °C for 2 min. The supernatant was discarded and the cells were resuspended in 1 mL of 1× PBS with 1 μg of RNAse and then incubated at 37 °C for 30 min. After RNase treatment, the cells were centrifuged and the supernatant was discarded. The cells were resuspended with 1× PBS and 1 μL of propidium iodide (10 μg/μL). Care was taken to protect the samples from exposure to sunlight. The DNA content of the cells was analyzed by flow cytometry (BD Accuri C6 Flow cytometer, BDBiosciences, Ann Arbor, MI, USA). The data was analyzed using Accuri software (BDBiosciences, Ann Arbor, MI, USA).

### 4.5. RNA Extraction and DNase Treatment

RNA extraction was performed by using a Qiagen RNAeasy mini prep kit according to the manufacturer’s instructions (Valencia, CA, USA). RNA was eluted in 30 µL RNase-free water and stored at −80 °C. Turbo DNase activation buffer (10%), 5 µg of RNA, and Turbo DNase (1 µL) were mixed and brought to 50 µL final volume with RNase-free water. The samples were incubated at 37 °C for 30 min followed by the addition of DNase inactivation reagent (10%) and incubated for 2 min at room temperature with occasional mixing. The samples were centrifuged for 1.5 min at 10,000 RPM, and the supernatant containing DNA-free RNA was transferred to fresh 1.5 mL tubes and stored at −80 °C.

### 4.6. Quantitative RT-PCR

RT-PCR was performed using a qScript-One step RT-PCR system (Quanta Biosciences, MD Cat. No. 95047). One hundred nanograms (100 ng) of DNase-treated RNA was used as template for one-step RT-PCR along with 3 µM forward and reverse primers (Table S5), 2× SYBRgreen buffer, 1 µL reverse transcriptase, and RNase-free water in 10 µL final volume. Amplification was performed for 40 cycles. Negative control RT-qPCR using primers targeting ATP1A1 (whose transcript was unchanged on the microarray following knockdown of Btf or TRAP150) showed less than a 0.1-fold change in cells treated with siRNA duplexes B1 and T4 after normalization to GAPDH. GAPDH *C*t values were subtracted from RT-qPCR values to determine Δ*C*_t_. ΔΔ*C*_t_ was calculated by subtracting the effect of control siRNA. Fold change was then determined by calculating 2^−ΔΔ*C*t^. The average of the treated samples were plotted relative to the average of controls to represent the fold change in expression following siRNA treatments. Standard deviation was calculated and shown as an error bar.

### 4.7. Global Transcript Analysis

Five replicates of the Human Exon Array 1.0 ST were performed using RNA samples isolated from five independent RNAi experiments (BtfsiRNA1, TRAP150siRNA4, or control siRNA) alongside SON-depleted samples as reported previously [21]. Each sample was hybridized and subjected to automated washing, staining, and scanning on the Fluidics Station 450 (Affymetrix) following fluidics protocol FS450-0001. Array results were analyzed using AltAnalyze [22] for differential gene expression in control versus Btf- or TRAP150-depleted samples as described in Ref. [21]. The primary data have been deposited in the NCBI GEO database under the accession number GSE102465.

### 4.8. Live Cell Microscopy

HeLa cells stably expressing H2B-YFP (gift from J. Swedlow, University of Dundee, Dundee, Scotland, UK) were used for live cell microscopy. Cells numbering 2 × 10^5^ were plated on a 60 mm coverslip for siRNA transfection with either control si2 targeting luciferase, Btf 1, TRAP150 si4, or Btf si1 and TRAP si4. Seventy-two hours (72 h) post-transfection, the coverslip with the cells was transferred to a live imaging chamber (FCS2; Bioptechs, Butler, PA, USA), perfused with L-15 medium (without phenol red) containing 20% fetal bovine serum and 5% penicillin/streptomycin and placed into a 37 °C environmental chamber on the stage of a Delta Vision RT imaging system. Different stages of mitosis were imaged by acquisition of *z*-stacks through the entire cell every 3 min for at least 2–3 h for samples treated with BtfSi1 or TRAPSi4 and for 10 min for at least 3–4 h for samples treated with BtfSi1 and TRAPSi4 (BT).

## 5. Conclusions

Our data show that Btf and/or TRAP150 depletion interferes with chromosome alignment and mitotic progression in human cells. Gene families regulated by Btf and TRAP150 are enriched for transcripts encoding cell cycle regulators, and many are misregulated in Btf/TRAP150 depleted conditions, pointing to control at the level of transcription or processing for cell cycle regulator transcripts as a critical component of mitotic progression pathways. In conclusion, our data provide new evidence that SR-like proteins are indispensable for the proper expression of cell cycle regulator transcripts and for maintaining normal mitotic progression.

## Figures and Tables

**Table 1:** Affymetrix HumanExon10ST Array results for differentially expressed major chromosomal passenger genes after Btf and TRAP150 depletion.

Transcript Name	Btf Si1		Btf Si2		TRAPSi4	
	Fold Change	*p*-Value	Fold Change	*p*-Value	Fold Change	*p*-Value
AURK-B	2.5	0.0002	1.7	0.003	2	0.001
CENP-E	2.8	0.003	2.5	0.027	3.0	0.012
CENP-F	4.4	0.0002	3.4	0.0015	4.1	0.01
BUB1	3.36	0.002	3.16	0.005	3.38	0.002
BUB1B	5.46	0.0012	3.3	0.003	3.5	0.002
CDK1	2.3	0.004	2.15	0.004	4.69	0.02
CDC-20	3.38	0.003	2.37	0.0002	5.8	0.02
MAD2L1	4.3	0.002	2.14	0.001	3.12	0.004

## Supplementary Materials

Supplementary materials can be found at www.mdpi.com/1422-0067/18/9/1956/s1.

## Abbreviations

Btf	Bcl-2 associated transcription factor-1; also called BCLAF
TRAP150	Thyroid hormone receptor-associated protein 150; also called THRAP3
SR protein	Serine-arginine rich protein
