# Supplementary material for: Alignment of Mitotic Chromosomes in Human Cells Involves SR-Like Splicing Factors Btf and TRAP150
Source: Int J Mol Sci. 2017 Sep 12;18(9):1956. doi: 10.3390/ijms18091956 (PMC5618605; doi:10.3390/ijms18091956)
Supplement: Supplementary file 1 [file ijms-18-01956-s001.pdf]

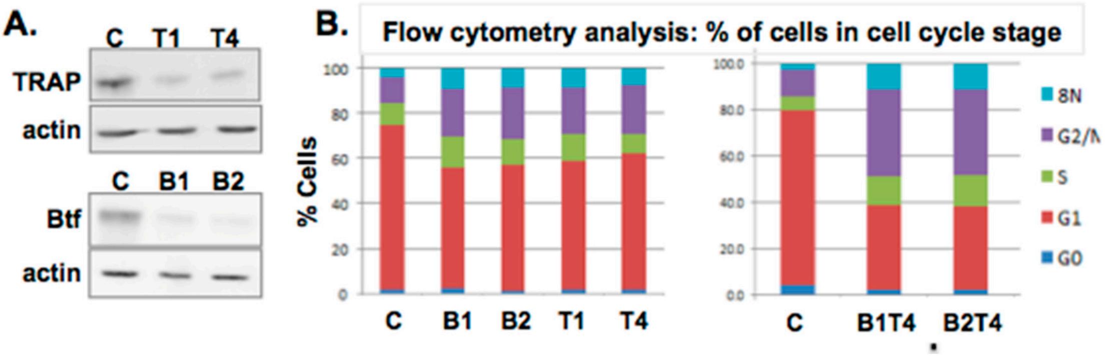

Supplemental Figure 1: Flow cytometry analysis of cells after independent (B1, B2, T1, T4) or combined (BT) depletion of Btf and TRAP150. (A) Immunoblot confirmed Btf and TRAP150 depletion for samples in (B). (B) Flow cytometry analysis of cells after independent (B1, B2, T1, T4) or combined (BT) depletion of Btf and TRAP150.

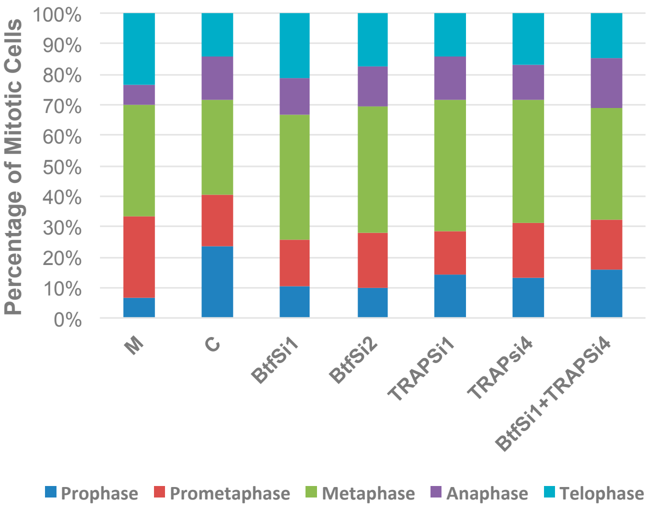

Supplemental Figure 2: Mitotic phase distribution of Btf and/or TRAP150 depleted cells. 500 mitotic cells were scored for mitotic phase per experimental condition in three replicate experiments (mock, controlSi, BtfSi1, BtfSi2, TRAPSi1, TRAPSi4, BtfSi1+TRAPSi4).

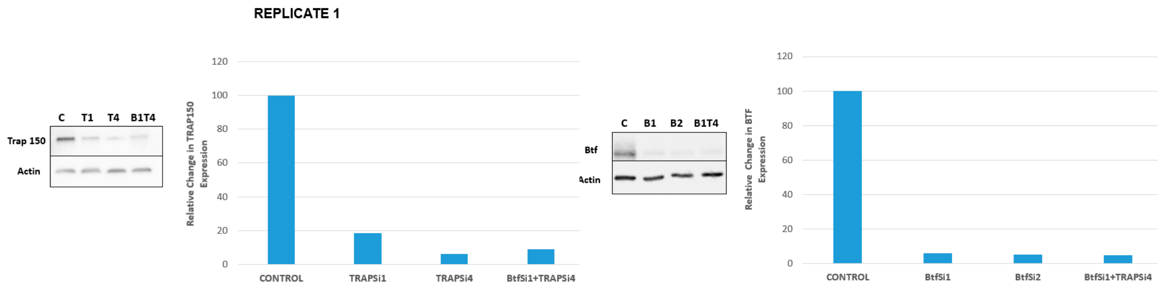

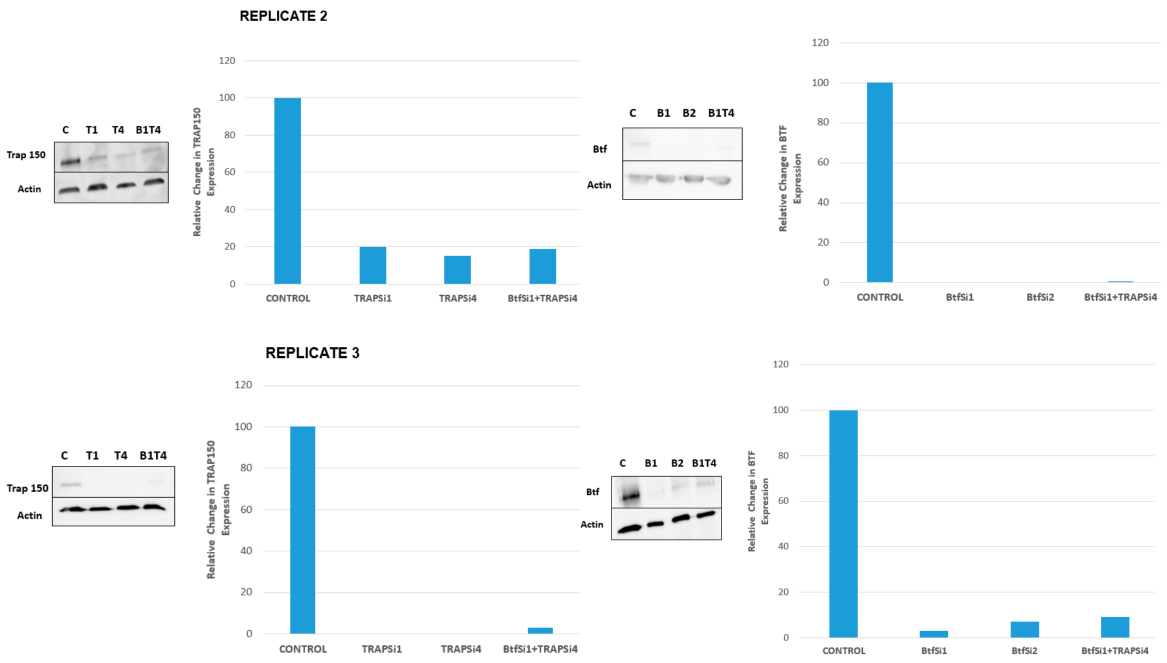

Supplemental Figure 3: Immunoblots show reduced expression levels of Btf or TRAP150 in three chosen out of five replicate RNAi samples used for RT-qPCR analysis validation of transcript abundance. Whole cell protein extracts from three replicate sets of HeLa cells transfected with control siGENOME2 targeting luciferase (C), Btf (B1, B2) or TRAP150 (T1, T4) were applied to SDS-PAGE and immunoblotted using antibodies against Btf or TRAP150. Actin was used as a loading control. Corresponding densitometry plots confirmed lower Btf and TRAP150 protein expression following treatment with siRNA duplexes targeting Btf and TRAP150 as compared to controls.

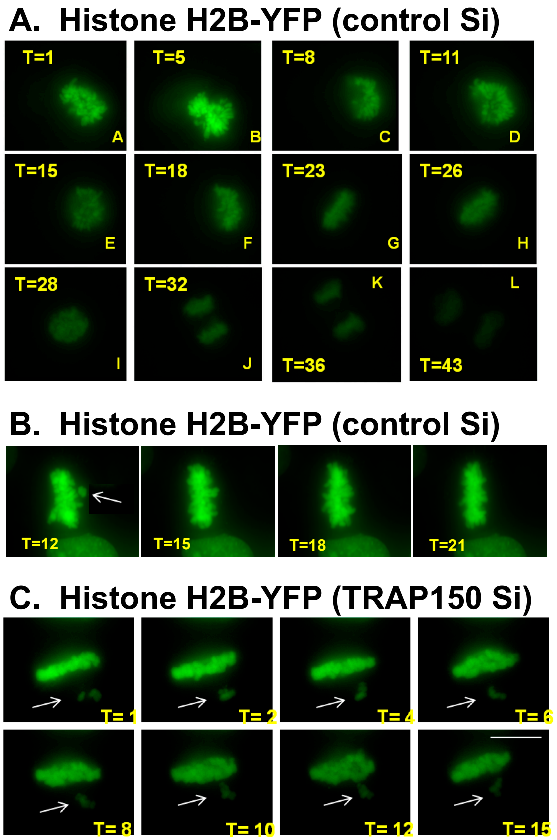

Supplemental Figure 4: Example of chromosome misalignment in TRAP150-depleted cells that shows data similar to observations in Btf-depleted cells (Figure 6). A. Cells treated with control siRNA duplexes show timing of mitosis from prophase (image A) to G1 (image L). B. Different images presented from the control cell in Figure 6 illustrate a metaphase cell treated with control siRNA duplexes having a briefly misaligned chromosome (arrow) that quickly aligns to the metaphase plate. (The first panel in this sequence (T=12) was captured 3 min prior to the panel in Figure 6b. The second panel T=15 also appears in Figure 6). C. Example from cells treated with TRAP150 si4 duplexes showing chromosome misalignment that persists for more than 15 minutes.

Supplemental Table 1: Top gene transcripts significantly upregulated after Btf depletion.

Symbol	Definition	Fold-change	t-test p-value
LMNB1	Lamin-B1	6.53	2.30e-05
EXO1	Exonuclease 1 (hExo1)	5.21	4.25e-05
XRCC2	DNA repair protein XRCC2	6.97	0.000187255
ASNS	Asparagine synthetase	5.14	0.000255689
POLR3G	DNA-directed RNA polymerase III subunit RPC7	5.04	0.000317388
SNORA58	Small nucleolar RNA SNORA58	6.05	0.000341396
MND1	Meiotic nuclear division protein 1 homolog	5.68	0.000378419
TMEM20	Transmembrane protein 20	6.38	0.000677746
C11orf82	Nitric oxide-inducible gene protein	8.23	0.000855792
SNRNP48	U11/U12 small nuclear ribonucleoprotein 48 kDa protein	5.11	0.000970151
FANCD2	Fanconi anemia group D2 protein	6.81	0.001133328
BUB1B	Mitotic checkpoint serine/threonine-protein kinase BUB1 beta	5.46	0.001218713
TRIP13	Thyroid receptor-interacting protein 13 binding protein)	5.64	0.001519661
C13orf3	Uncharacterized protein C13orf3	5.64	0.001700366
SNORD77	Small nucleolar RNA SNORD77	5.17	0.002691979
DEPDC1	DEP domain-containing protein 1A	8.69	0.007388267
KLHL17	Kelch-like protein 17 (Actinfilin)	5.53	0.008181216
PLK1	Serine/threonine-protein kinase PLK1	6.75	0.017195056

Supplemental Table 2: Top gene transcripts significantly downregulated after Btf depletion.

Symbol	Definition	Fold-change	t-test p-value
VTN	Vitronectin Precursor (Serum-spreading factor)	-8.01608	1.62e-07
FXYD3	FXYD domain-containing ion transport regulator 3 Precursor	-6.21169	1.91e-07
CYFIP2	Cytoplasmic FMR1-interacting protein 2 (p53-inducible protein 121)	-12.5107	3.39e-07
EHF	Epithelium-specific ETS homologous transcription factor (hEHF)	-13.6964	1.57e-06
CDH5	Cadherin-5 Precursor (Vascular endothelial cadherin)(VE-cadherin)	-6.93162	3.04e-06
APOD	Apolipoprotein D Precursor (Apo-D)	-8.00802	4.19e-06
ST6GAL1	Beta-galactoside alpha-2,6-sialyltransferase 1	-61.1597	5.71e-06
SULF2	Extracellular sulfatase Sulf-2 Precursor	-6.49092	6.51e-06
P2RY6	P2Y purinoceptor 6 (P2Y6)	-6.46645	2.77e-05
AC008734.7	Putative uncharacterized protein	-6.4416	3.70e-05
PGM2L1	Glucose 1,6-bisphosphate synthase (EC 2.7.1.106)(Phosphoglucomutase-2-like 1)(PMMLP)	-5.65665	0.000125
IL6	Interleukin-6 Precursor (IL-6)(B-cell stimulatory factor 2)	-5.49985	0.000171
SERPINE1	Plasminogen activator inhibitor 1 Precursor (PAI-1)(PAI)	-5.95979	0.000241
TUBA1A	Tubulin alpha-1A chain (Tubulin B-alpha-1)(Tubulin alpha-3 chain)(Alpha-tubulin 3)	-5.29916	0.000284
SPINK4	Serine protease inhibitor Kazal-type 4 Precursor (Peptide PEC-60 homolog)	-6.4291	0.000351
LXN	Latexin (Endogenous carboxypeptidase inhibitor)(ECI)(Tissue carboxypeptidase inhibitor)(TCI)(MUM)	-6.27317	0.000626
GPR87	Probable G-protein coupled receptor 87	-7.40938	0.000628
GPR141	Probable G-protein coupled receptor 141	-6.19249	0.000699
C1S	Complement C1s subcomponent Precursor (C1 esterase)	-5.72968	0.000995
TMEM45B	Transmembrane protein 45B	-8.06361	0.001295

Supplemental Table 3: Top gene transcripts significantly upregulated after TRAP150 depletion.

Symbol	Definition	Fold-change	t-test p-value
FKBP7	FK506-binding protein 7 Precursor	6.559537817	0.0003
PLSCR4	Phospholipid scramblase 4 (Cell growth-inhibiting gene 43 protein)	5.980172085	0.0002
LEPROT	Leptin receptor gene-related protein	5.957780616	0.0022
TXNDC12	Thioredoxin domain-containing protein 12 Precursor	4.993297679	0.0025
ACTC1	Actin, alpha cardiac muscle 1 (Alpha-cardiac actin)	4.854396947	0.0002
PARP14	Poly [ADP-ribose] polymerase 14 (PARP-14)	4.699940524	0.0004
KIF20A	Kinesin-like protein KIF20A (Rabkinesin-6)	4.681067704	0.0009
LMNB1	Lamin-B1	4.656025496	0.0019
FBLN7	Fibulin-7 Precursor (FIBL-7)	4.632983893	0.0004
SGOL2	Shugoshin-like 2 (Tripin)	4.592271552	0.0018
FBLN5	Fibulin-5 Precursor	4.393203514	0.0001
LPL	Lipoprotein lipase Precursor	4.231539108	0.0011
DTL	Denticleless protein homolog (Retinoic acid-regulated nuclear matrix-associated protein)	4.168649282	0.0002
CDK2	Cell division protein kinase 2 (p33 protein kinase)	4.149234657	0.0007
CENPF	centromere protein F	4.14713571	0.0003
CYBRD1	Cytochrome b reductase 1	4.126196421	0.0002
KIF11	Kinesin-like protein KIF11 (Thyroid receptor-interacting protein 5)	4.088121054	0.0010
C8orf84	RPE-spondin Precursor	4.053769804	7.50e-05
DUSP2	Dual specificity protein phosphatase 2	3.971877835	5.63e-05

Supplemental Table 4: Top gene transcripts significantly downregulated after TRAP150 depletion.

Symbol	Definition	Fold-change	t-test p-value
CYFIP2	Cytoplasmic FMR1-interacting protein 2 (p53-inducible protein 121)	-9.075634443	6.18e-05
IL6	Interleukin-6 Precursor (IL-6)(B-cell stimulatory factor 2)	-7.566821958	8.46e-05
SERPINE1	Plasminogen activator inhibitor 1 Precursor	-6.589847138	8.14e-06
EHF	Epithelium-specific Ets homologous factor (hEHF)	-5.792819986	3.99e-05
FXYD3	FXYD domain-containing ion transport regulator 3 Precursor	-4.501393112	4.92e-07
ANPEP	Aminopeptidase N (hAPN)(Alanyl aminopeptidase)	-3.738339255	3.06e-05
MYO1G	Myosin-Ig [Contains Minor histocompatibility antigen HA-2(mHag HA-2)]	-3.390153075	1.73e-05
SUSD2	Sushi domain-containing protein 2 Precursor	-3.357988214	3.73e-07
TINAGL1	Tubulointerstitial nephritis antigen-like Precursor	-2.892039987	6.29e-05
A2M	Alpha-2-macroglobulin Precursor (Alpha-2-M)	-2.70629292	5.10e-05
ITGB4	Integrin beta-4 Precursor (GP150)(CD104 antigen)	-2.672373516	1.28e-06
ABCA12	ATP-binding cassette sub-family A member 12	-2.582978241	8.00e-06
LGALS9C	Galectin-9C (Galectin-9-like protein B)	-2.577175214	7.06e-05
CCDC64	Coiled-coil domain-containing protein 64A	-2.571150505	6.87e-05
SNX29	Sorting nexin-29	-2.392916408	1.91e-05
FAIM2	Fas apoptotic inhibitory molecule 2	-2.253138614	8.28e-06
TMEM40	Transmembrane protein 40	-2.174429725	4.11e-05
PTK6	Tyrosine-protein kinase 6 (Breast tumor kinase)	-2.090302396	3.19e-05
CCND1	G1/S-specific cyclin-D1 (PRAD1 oncogene)(BCL-1 oncogene)	-2.079302091	5.97e-05

Supplemental Table 5: Primer sequences for RT-qPCR of mitotic regulator transcripts.

AURKB for<br>AURKB rev	GGCAAGAGAAAAGCAAAGCA<br>TCTTCTGAGCTGATGCTCCA
CENP-F for<br>CENP-F rev	GTGGCAACAGAAGCTGACAA<br>TCTTCTGTGTCGATGCCAAG
BUB1 for<br>BUB1 rev	AACTGAGCGCCATGTCTT<br>CCAAAGGAACAACAGGA
CDK1 for<br>CDK1 rev	GCCTTCTAATATCTCCCTTCG<br>GCCATTCAATCGCTACCTCAT
CDC-20 for<br>CDC-20 rev	ATGTGTGGCCTAGTGCTCCT<br>TGATGCTGGGTGAATGTCTG
MAD2L1 for<br>MAD2L1 rev	GTTCTTCTCATTCGGCATCAACA<br>GAGTCCGTATTTCTGCACTCG
